# Recent advances and insights into the bioactive properties and applications of *Rosa canina* L. and its by-products

**DOI:** 10.1016/j.heliyon.2024.e30816

**Published:** 2024-05-07

**Authors:** Oana-Raluca Negrean, Anca Corina Farcas, Silvia Amalia Nemes, Diana-Elena Cic, Sonia Ancuta Socaci

**Affiliations:** a Department of Food Science, Faculty of Food Science and Technology, University of Agricultural Sciences and Veterinary Medicine Cluj-Napoca, 400372, Cluj-Napoca, Romania; b Life Science Institute, University of Agricultural Science and Veterinary Medicine of Cluj-Napoca, Cluj-Napoca, Romania

**Keywords:** Rosehip, Antioxidants, Health benefits, Bioactive compounds, Functional products, Pharmaceuticals

## Abstract

Rosa canina L., commonly known as rosehip, is of notable scientific interest for its applications in nutrition, cosmetics, and pharmaceuticals. This review article highlights its health-promoting properties, including antioxidant, anti-inflammatory, hepatoprotective, and anticarcinogenic effects, attributed to its rich content of phenolic acids, carotenoids, tocopherols, and vitamins. With growing interest in sustainable practices, rosehip by-products are increasingly valorized. For instance, cold-pressed rosehip seed oil is a valuable source of polyunsaturated fatty acids, while incorporating rosehip pomace into snacks enhances their nutritional profile, positioning them as potential functional foods and dietary supplements. This article aims to provide a comprehensive overview of advancements in utilizing rosehip and its by-products, emphasizing their role in enriching food and pharmaceutical products with nutritional and functional bioactivities.

## Highlights

• Rosehip represents a sustainable source of valuable bioactive compounds.
• Strategies for reusing rosehip by-products can be applied to create new products.
• Rosehip has multifaceted applications in nutrition and pharmaceuticals.
• Exploitation of rosehip yields nutritional, functional, and economic advantages.

## 1 Introduction

Rosehip *(Rosa canina* L.*)* belongs to the *Rosa* genus of the *Rosaceae* family and is growing wildly around the globe in areas such as Asia, North America, Europe, Middle East [1,2]. Various parts of this plant have been traditionally used for medicinal and culinary purposes for centuries. European folk medicine uses rosehip in the treatment of gastrointestinal disorders, infections, and fever [3] Rosehip is a nutritionally valuable fruit containing substantial amounts of bioactive compounds, such as vitamins, minerals, antioxidants, and phenolic compounds, which contribute to its health-promoting properties. The main bioactive compounds of rosehip are phenolic acids (gallic acid, ellagic acid, caffeic acid, *p*-coumaric acid), carotenoids (lycopene, β-carotene, zeaxanthin), anthocyanins, tocopherols (α- and β-tocopherols), tannins [4,5] and flavonoids (flavan-3-ols) [6].

One of the most well-known benefits of rosehips is their ability to boost the immune system. This is due to their high content of vitamin C, which has been shown to enhance immune function by increasing the production of white blood cells and antibodies [7]. Also, rosehips are an important dietary source of vitamins, such as A, E, and K, and minerals, including calcium, magnesium, potassium, and phosphorus, which contribute to their nutritional value [8]. The antioxidant capacity of rosehips cannot be overlooked either. Antioxidants are essential for combating oxidative stress, a process linked to the development of various chronic diseases and aging. The diverse range of antioxidants present in rosehips, including flavonoids and carotenoids, effectively neutralize harmful free radicals, thereby minimizing cellular damage and promoting overall well-being [9]. Furthermore, the presence of antioxidants in rosehips confers protective effects against inflammation, a key factor implicated in the onset and progression of chronic diseases like cancer, diabetes, and cardiovascular ailments. By inhibiting the reactive oxygen species and mitigating inflammatory processes, rosehips demonstrate their potential as a natural alternative in combating the above-mentioned debilitating conditions [10]. Research has also demonstrated that rosehips may have potential benefits for skin health. The high levels of vitamin C in rosehips are important for collagen synthesis, which is essential for maintaining healthy skin [11]. Furthermore, the phenolic compounds present in rosehips have been shown to have anti-inflammatory [12,13] and anti-aging effects on the skin [14].

Every year, the entire food chain generates around 1.3 billion tons of food loss and waste [15]. To address global hunger concerns, researchers have directed their attention toward the valorization of by-products from the food industry. Regarding rosehip, studies have primarily focused on the valorization of its seeds and pomace. Cold-pressed rosehip seed oil is a valuable source of bioactive compounds, including mono/polyunsaturated fatty acids (oleic and linoleic acids) [16], [17] and carotenoids, lycopene, phenolics, vitamin C, tocopherols, squalene, and chlorophyll [18].

When it comes to the extraction of bioactive compounds from rosehip, a multitude of techniques are employed. These range from conventional solvent-based methods, which utilize basic equipment, to advanced procedures such as microwave-assisted extraction, ultrasound-assisted extraction, and enzyme-assisted extraction [19]. Parameters such as extraction duration, temperature, solvent-to-sample ratio, and the type of solvent used are critical factors influencing the efficiency and selectivity of extraction. Additionally, supplementary purification steps may be necessary to remove undesirable compounds [20].

The potential applications of rosehips in the food industry make them a promising ingredient for the development of functional products that promote health and wellness. In this regard, both the rosehips and their by-products offer a wide range of potential applications in functional foods and dietary supplements, in addition to the production of conventional products such as juices, jams, and teas. Notably, the incorporation of rosehip flour in bread dough has led to a significant increase in the bread's nutritional value and improved its rheological properties, with a positive correlation observed with the amount of rosehip flour used [21]. Also, incorporating rosehip pomace into snack composition has been shown to significantly enhance their total phenolics and vitamin C content, suggesting the potential of the by-product to be used as a functional ingredient [22]. Furthermore, the diverse bioactivities exhibited by rosehips render them a highly promising ingredient for the formulation and development of nutraceuticals, cosmetics, and pharmaceutical products.

This review article aims to provide a comprehensive overview of the advancements made in the exploitation of rosehip and by-products, while also addressing its potential contribution to enhancing the nutritional value and functional bioactivities of food and pharmaceutical products. The originality of this approach lies in the comprehensive exploration of rosehips and their by-products as a source of bioactive compounds with multifaceted applications. The review not only consolidates existing knowledge but also emphasizes the need for further research in several key areas.

**Fig. 1:**
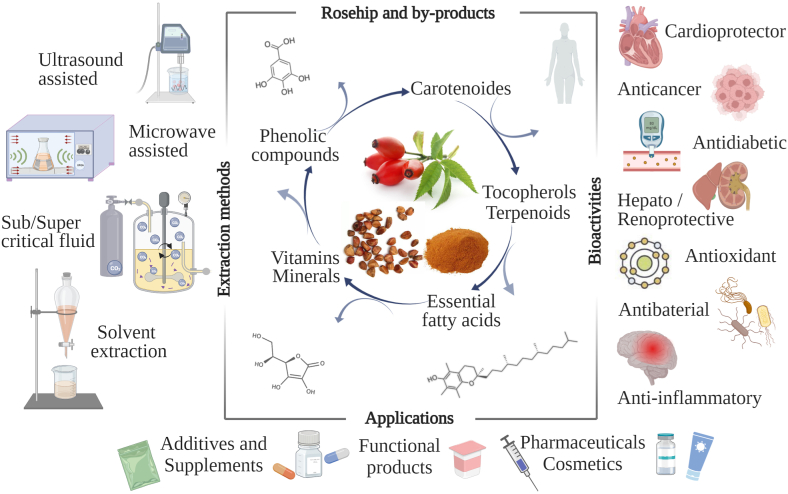
Schematic overview of the main extraction technologies, bioactivities, and applications of rosehips and by-products.

To fully maximize the positive properties of rosehips, it is essential to consider several aspects related to processing and extraction technologies in order to maintain their nutritional and biologically active effects while also ensuring the sustainability of developing various value-added products. These important aspects, which will be detailed in the following sections, are systematically highlighted in Fig. 1.

## 2 Rosehip's bioactive compounds and their impact on human health

This section seeks to delve into the intricate details of these compounds and their multifaceted effects on health and well-being. The focus of this section will be on the diverse array of bioactive constituents found in rosehips, such as polyphenols, carotenoids, vitamins, tocopherols, essential lipids, and minerals. Rosehip represents a valuable natural source abundant in bioactive compounds whose potential as a functional ingredient and a medicinal plant demands further scientific exploration to enhance human health and well-being [23]. The rosehip plants includes a spectrum of phenolic compounds, with tannins and flavonoids like kaempferol, rutin, and quercetin being particularly notable. Phenolic acids, including ellagic acid and gallic acid, along with anthocyanins such as cyanidin-3-glucoside, further enrich this complex phytochemical profile. Beyond these, rosehips are also rich in carotenoids, including lycopene and beta-carotene, and is an excellent source of vitamin C, which synergizes with flavonoids to exhibit potent antioxidant activity [24].

The efficacy of rosehip fruits' medicinal properties is intricately linked to the quality and quantity of compounds endowed with potent antioxidant capabilities, making them a crucial factor in understanding their therapeutic potential [25]. Butkevičiūtė et al. (2022) employed the both spectrophotometric and chromatographic techniques to determine and identify representative phenolic and flavonoid compounds in rosehip fruit samples. Their findings revealed that the total amount of phenolic compounds ranged from 10.89 mg GAE/g to 26.49 mg GAE/g. The HPLC technique was utilized for both qualitative and quantitative analysis, revealing the presence of flavan-3-ol group compounds such as catechin (ranging from 26.30 ± 1.31 μg/g to 522.48 ± 26.12 μg/g), (−)-epicatechin (ranging from 2.12 ± 0.11 μg/g to 20.66 ± 1.03 μg/g), procyanidin B1 (ranging from 1.65 ± 0.08 μg/g to 40.89 ± 17.04 μg/g), and procyanidin B2 (ranging from 5.54 ± 0.28 μg/g to 86.95 ± 4.35 μg/g) [26].

The main carotenoids found in rosehips are lutein, zeaxanthin, β-carotene, and lycopene, also responsible for the characteristic red color [9]. The concentration of lycopene in rosehips is affected by various factors, such as fruit ripeness, soil quality, and environmental conditions [3,27]. Harvest time also impacts the carotenoid content, with earlier-harvested rosehips displaying lower concentrations compared to those harvested at the end of the season [7]. From a quantitative perspective, rosehips contain lutein and zeaxanthin in proportions ranging from 12.89 % to 20.53 % and 45.57 %–70.34 %, respectively, of the total carotenoid content. Additionally, beta-carotene accounts for 54.79 %–77.31 % of the identified total carotenoids [12]. Notably, *R. rugosa* and *R. canina* have significantly higher (all-E)-lycopene concentrations ranging from 7.4 to 7.9 mg/100 g compared to other common sources [27,28].

Rosehip oil is highly valued for its functional, tocopherols (α-tocopherol and γ-tocopherol), sterols (e.g., campesterol, β-sitosterol), and essential fatty acids (e.g., linoleic acid and α-linolenic acid) [29,30]. The tocopherol levels can reach 1124.2 mg/kg [30], while oleic acid (C18:1), linoleic acid (C18:2), and linolenic acid (C18:3) are the predominant unsaturated fatty acids found in rosehip cold-pressed oils [31].

Despite containing a wide variety of vitamins (A, E, K and B1), rosehips stand out particularly due to their high content of ascorbic acid. The greatest concentration of vitamin C is typically found in mature, hardened rosehips ranging from 400 to 1500 mg/100 g, far surpassing the amounts found in apples and currants [[32], [33], [34]].

### 2.1 Antioxidant activity

Along the years, it was mentioned that the high antioxidant activity values of rosehip extracts are due to a strong interaction between polysaccharides and organic acids, and most important with phenolic antioxidants (flavonoids and phenolic acids) [35]. Health problems such as bronchitis, rheumatism, and type II diabetes are the result of excessive production of free radicals, but plants rich in antioxidants are inhibiting and scavenging free radicals [36]. A wide range of functions in the body happen because of the vitamins, including coenzymes activity, precursors activity, antioxidative effect, calcium and phosphorus uptake regulation, and coagulation regulation [9]. Rosehip is known to contain the highest amount of vitamin C among other fruits, which could be explanatory for their antioxidant activity. Furthermore, rosehip is rich in carotenoids (lycopene, β-carotene, zeaxanthin) [37], bioactive compounds bounded to the antioxidant activity. Tannins, flavonoids, and phenolic acids were discovered to be a very important group of biologically active compounds found in rosehip; these compounds being known also for their antioxidant properties [38].

There are several *in vitro* assays available to assess the antioxidant capacity of food and biological samples. The sensitivity of these methods is affected by several factors, including pH and the presence of lipophilic and/or hydrophilic compounds [39]. The usual method used to determine antioxidant activity it's ferric reducing ability of plasma (FRAP), a simple and low-cost method, assay used by Kocza et al. (2018) to determine the antioxidant capacity of some *Rosa* species, including *Rosa canina* L. (rosehip). Water and ethanol extracts were tested for antioxidant capacity, FRAP values ranging from 123.8 mmol to 314.4 mmol AA/g (expressed as ascorbic acid equivalents per g dry weight) in water extracts. Rosehip water extracts presented the second highest FRAP values, after *R. spinosissima* water extract. In the same manner, ethanolic extracts of *R. spinosissima* and *R. canina* had significantly high antioxidant properties [8]. According to Kubczak et al. (2020), the leaf and twig ethanolic extracts demonstrated significant effects on various markers of oxidative stress. Notably, the lipid peroxidation level showed a significant decrease when the highest concentration of 50 μg/mL was applied. Interestingly, the extracts did not exhibit any notable influence on thiol group oxidation. However, both leaf and twig extracts, when used at higher concentrations, exhibited a substantial reduction in protein carbonylation. Additionally, the application of 5 μg/mL extracts resulted in a remarkable decrease in DPPH free radical activity [4].

The antioxidant capacity of rosehip decoctions was also tested by DPPH, ABTS and Ferrous ion chelating assays. Orhan et al. [40] demonstrated that rosehip decoctions had strong inhibitor activity on ABTS and DPPH but even though they did not contain vitamin C. DPPH radical scavenging activity of all samples increased with concentration. At 2000 μg/mL, samples exhibited higher or similar antioxidant activity with BHT (control sample). On the other hand, rosehip samples did not exert any ferrous ion chelating effect [40]. Gherghina et al. (2018) conducted a study evaluating how storage methods of the fruits could affect bioactive nutrients while preserving the antioxidant capacity of them is very important. Carotene, ascorbic acid and total polyphenols were determined using spectrophotometrically methods. Bioactive compounds determined from rosehip fruits decreased after six months of storage, regardless of storage conditions, but polyphenols were found to be more stable than the other analyzed compounds (carotene and vitamin C). Although, fruits stored in frozen conditions presented better results in terms of antioxidant content [41] Another study conducted by Strugała et al. (2016) highlighted the antioxidant activity of four ethanolic fruit extracts (blackcurrant, chokeberry, hawthorn, and rosehip) and their mixture with linseed oil on a model lipid membrane. Rosehip and chokeberry presented the highest antioxidant activities [42]. Gingerbread enriched with rosehip was tested *in vitro* for their antioxidant activity, using DPPH assay in the conditions of gastric digestion. Pepsin was used to simulate gastric digestion. The “Visocovitaminnii” rosehips variety is distinguished by their high antioxidant activity and high level of polyphenols, of 5484 mg GAE/100 g, of which 1199 mg GAE/100 g are flavonoids. Gingerbread's physical, chemical, and sensory qualities were improved by rosehip pulp of 2 % and 4 %, which also ensured antioxidant activity and microbiological stability by lowering total viable count [43]. Antioxidant properties of rye starch films containing rosehip ethanol extract were measured with ABTS and DPPH assays. Different concentrations of rosehip extract (0.4, 0.7, and 1.0 g per 100 mL) were incorporated into the films. Rosehip extract 1.0 % films exhibit the highest ABTS and DPPH radical scavenging activities (96.87 % and 80.22 %, respectively). There were no significant differences between film containing 0.7 % (95.95 %) and 1.0 % (96.87 %) rosehip extract during ABTS radical scavenging activity, suggesting that the rosehip extracts antioxidant properties have persisted even after the films have been formed [44]. Alzamara et al. (2017) tested the antioxidant activity of rosehip marmalade yogurt (5, 10 and 15 % marmalade). After 21 days of cold storage, the rosehips marmalade yogurt's antioxidant activity and phenolic component concentration were assessed and compared to plain yogurt. The yogurt's ability to scavenge radicals was strengthened by the rosehips marmalade which remained richer in antioxidant and phenolic compounds when compared to plain yogurt, even though significant declines in antioxidant activity and phenolic component concentration occurred during the time of cold storage [45]. Güney (2020b) determined antioxidant activity of ethanolic rosehip seeds extract, obtaining a DPPH radical scavenging capability of 130.64 mg TE/100 g [46]. According to the findings of Tanor et al. in 2020, the ethanolic extract of rosehip seed press cake demonstrated antioxidant activity with radical scavenging percentages ranging from 10.32 % to 76.06 %. These results were observed at different concentrations of total phenolics and flavonoids, ranging from 200 μg/mL to 3000 μg/mL. In a different study, the IC50 value of the ethanol seed extract was determined to be 1367.06 μg/mL. Comparatively, the IC50 value of ascorbic acid, a known antioxidant, was found to be 200 μg/mL. These results indicate that the ethanol seed extract's antioxidant activity is less potent than ascorbic acid, as higher concentrations (greater than 1000 μg/mL) of the extract are required to achieve a significant effect [47].

### 2.2 Antidiabetic effect

High levels of blood glucose are characteristic of a group of metabolic diseases called diabetes mellitus. Insulin-dependent diabetes (type 1) and non-insulin-dependent diabetes (type 2) are both chronic conditions. Rosehip fruits have been shown to contain volatile compounds, phenolic compounds, and other antioxidant compounds that may be effective in the treatment of these disorders [48]. Asghari et al. (2015) conducted research focused on α-glucosidase inhibitors from rosehip fruits, this enzyme being responsible for the final step in the hydrolysis of carbohydrates in the digestive system. *Rosa canina* fruit extracts were tested for their inhibitory effect on α-glucosidase activity using *n*-hexane, ethyl acetate, acetone, and methanol extracts. The acetone extract had the highest inhibitory activity against α-glucosidase (IC_50_ 0.3 g/mL), followed by the methanol extract (IC_50_ of 0.5 g/mL) [49].

Antidiabetic and antihyperlipidemic of rosehip ethanol extracts presented an interest of study for Taghizadeh et al. (2016). The results showed that the oral administration of rosehip fruit extract significantly ameliorated the high levels of blood glucose in streptozotocin induced diabetic rats compared to the control group (fed with distilled water). Moreover, serum triglyceride levels significantly decreased by the administration of rosehip extract compared with control [50]. Compared to Taghizadeh et al. (2016), another study observed the effects of rosehip on glycemia and lipid profile on humans with type 2 diabetes, aged 35–60 years with fasting blood glucose levels between 130 and 200 mg/dL and HbA1c (hemoglobin A1c) between 7 and 9 %. Two groups of patients were formed, one received 750 mg rosehip fruit extract and the other one 750 mg toast powder as placebo two times a day respectively for three months. When compared to the baseline, the fasting blood glucose level in the rosehip group decreased significantly after 3 months [51]. Furthermore, when compared to the baseline, total cholesterol/HDL-C levels were significantly lower in the *R. canina* group. Other blood parameters did not change significantly during the study when compared to placebo and baseline [51].

Rosehip's effect on glucose metabolism in HepG2, a tumor cell line, was also studied [52]. The extract's effect on glucose diffusion across the dialysis membrane, a convenient model for assessing cellular glucose absorption, was observed as well. Fattahi et al. (2017a) findings confirmed that rosehip extract can act as a growth factor for pancreatic b-cell lines, providing a novel mechanism for the observed anti-diabetic effect [52].

Prediabetes represents a high-risk state for developing diabetes with a reduced tolerance to glucose but normal fasting blood glucose levels is becoming more common around the world. Chen et al. (2017) focused their research on anti-prediabetic effect of rosehip extract. Long-term supplementation with rosehip extract managed to improve the tolerance to glucose, decreased secretion of insulin, and preserved pancreatic beta-cells function in SDT (spontaneously diabetic Torii) rats at the pre-diabetic stage, preventing or delaying the onset of diabetes [53].

As a conclusion, rosehip extract's beneficial effects in diabetes management are linked to its ability to modulate cell signaling pathways involved in glucose metabolism and insulin sensitivity, ultimately influencing the overall metabolic state and reducing the risk of diabetes progression. This includes both direct effects on enzyme inhibition and indirect effects on cell signaling that enhance pancreatic function and improve insulin sensitivity.

### 2.3 Anti-inflammatory effects

It is suggested that an uncontrolled inflammatory response is the primary cause of a wide range of disorders, including allergies, cardiovascular dysfunctions, metabolic syndrome, cancer, and autoimmune diseases, placing a tremendous economic burden on individuals and, as a result, on society. Inflammation is the body's protective response to harmful stimuli, such as allergens and/or damage to the tissues [54]. Inflammatory diseases and the problems they are associated with have been treated using plants or products derived from plants since ancient. Flavonoids are an example of a polyphenolic compounds that can block transcription factors or regulatory enzymes involved in the inflammatory process [55]. The presence of phenolic compounds in rosehip represents the main reason why rosehip has an anti-inflammatory effect.

Anti-inflammatory effect of fruit extracts from blackcurrant, chokeberry, hawthorn, and rosehip was studied by Strugała et al. (2016). The results showed that the tested fruit extracts may have anti-inflammatory properties, as evidenced by the inhibition of COX-1 (cyclooxygenase-1) and COX-2 (cyclooxygenase-2) enzyme activity (Strugała et al., 2015). By oxidizing arachidonic acid to endoperoxides at the organismal level, these enzymes offer substrates for prostaglandin, prostacyclins, and thromboxanes, which are key mediators of the inflammatory process. The highest inhibition of the two enzymes was presented by blackcurrant ethanol extract (77.6 and 70.5 %, respectively) followed by rosehip ethanol extract (64.9 and 72.5 %) [42].

Furthermore, the anti-inflammatory activity of rosehip aqueous extract was tested in animal models. Rats' paws inflammation was induced by being injected with formalin, and the effectiveness of the extract's treatment was evaluated. Quantities of 100, 300, and 700 mg/kg of the extract were dosed. Also, normal saline and sodium salicylate were given to the negative and positive control groups. The aqueous extract of rosehip reduced the inflammation caused by formalin during the acute and chronic phases of inflammation over a 7-day period. According to the findings of this study, aqueous extract of rosehip has anti-inflammatory properties [56].

### 2.4 Antibacterial effects

To ensure the availability of nutritious and safe food for consumers, researchers worldwide are actively investigating and evaluating antimicrobial compounds that can effectively inhibit the growth of pathogenic bacteria in food. Rovná et al. [57] study aimed to assess the antimicrobial properties of ethanolic rosehip fruit extract. According to the results, the minimum inhibitory concentration (MIC) of rosehip fruit extracts against *E. coli* was the lowest (32 g/mL), followed by *K. pneumonia* at 64 g/mL, being concluded that the fruit ethanolic extract has antimicrobial activity against *E.coli*. Moreover, rosehip flower extract was tested against microorganisms by another study. Three microscopic filamentous fungi strains, *Aspergillus niger*, *Fusarium culmorum*, and *Alternaria alternata*, as well as two Gram-negative bacteria, *Escherichia coli* and *Pseudomonas aeruginosa*, were tested against ethanolic and methanolic extracts of several flowers using the agar well diffusion method. Rosehip flower ethanolic extract had the best antibacterial activity against *Pseudomonas aeruginosa* and against *Escherichia coli* [57].

Herbal teas are the most often used natural alternatives for the treatment of infectious diseases, and they are currently gaining importance as a consequence of increasing antibiotic resistance. Rosehip was one of 31 herbal teas Hacioglu et al. [58] tested for their antimicrobial properties against some common and clinical isolates of *Pseudomonas aeruginosa*, *Acinetobacter baumannii*, *Escherichia coli*, *Klebsiella pneumoniae*, *Enterococcus faecalis*, methicillin-susceptible/resistant *Staphylococcus aureus*, and *Candida albicans.* The disk diffusion and microbroth dilution procedures were used to determine the antibacterial properties of the teas, and the time killing curve and microbroth checkerboard methods were used to investigate the combination studies. The findings indicated that black tea, green tea, and rosehip bag teas could be used as safe and efficient adjuvants to ampicillin treatment for bacterial infections, but rosehip and pomegranate blossom with ciprofloxacin or cefuroxime, have antagonistic interactions [58].

### 2.5 Anticancer and antiproliferative effects

The second cause of death worldwide is cancer, the development of cancer being caused by a succession of gene changes that alter how cells operate [59]. Over the years, scientists have directed their attention to find safe and effective methods on treating or preventing cancer, studying plants rich in bioactive compounds that were demonstrated to have anticancer or antiproliferative activity.

The goal of the Berkoz et al. (2019) study was to assess and screen the impact of apoptosis and the anticancer potential of rosehip ethanolic extract on human breast cancer cell lines, MCF-7 and MDA-MB-468. Rosehip ethanol extract's anti-proliferative ability was assessed using MTT (3-(4,5-dimethylthiazolyl-2)-2, 5-diphenyltetrazolium bromide), flow cytometry, annexin V/PI double staining, and caspase-3 activity. The results of MTT revealed that the ED_50_ of both human breast cancer cell lines was 25 μg/mL of rosehip extract, 48 h after treatment. Rosehip extract caused MCF-7 to undergo late apoptosis and MDA-MB-468 to undergo early apoptosis, according to flowcytometry using annexin V/PI. The results of the study showed that the ethanol extract of rosehip had a cytotoxic effect on the MCF-7 and MDA-MB-468 cancer cell lines depending on the dose of exposure without affecting normal cells [60].

Rosehip extracts have been shown to have antiproliferative and antioxidant properties in Caco-2 human colon cancer cells. The samples that were evaluated were total extract, vitamin C, neutral polyphenols, and acidic polyphenols. All the extracts showed substantial cytotoxicity after 72 h, at both low and high doses. When cells were exposed to hydrogen peroxide in the presence of plant fractions, ROS (Reactive Oxygen Species) production was reduced significantly. As a result, Jiménez et al. 2016 data demonstrated that rosehip extracts are a potent antioxidant that inhibits cell proliferation in Caco-2 cells [61].

The anti-metastatic and anti-cancer activity of biosynthesized silver nanoparticles from rosehip extract on the human colon adenocarcinoma cell line HT29 was investigated by Aydin Acar & Pehlivanoğlu, (2019). For 48 h, HT29 cells were incubated with various concentrations of AgNPs (0–20 g/mL). The MTT assay was used to investigate HT29, and the IC_50_ value was found to be 7,89 g/mL after 48 h of incubation. R-AgNPs' anti-metastatic potential was tested on HT29 cells using a scratch assay. Cell motility was significantly reduced by RAgNPs in a dose-dependent manner. The concluded results showed that the biosynthesized AgNPs may be promising new therapeutic agents for the treatment of human colon cancer [62].

### 2.6 Protective effects against kidney diseases

The effects of hydroalcoholic extract of rosehip fruit were studied on nephrolithiasic rats with the purpose of evaluating the possible therapeutic potential of the extracts. For 30 days, five groups of ten rats each received tap water (group I), 1 % EG (ethylene glycol) (group II), 250 mg/kg RC (*Rosa canina*) + 1 % EG (group III), 500 mg/kg RC + 1 % EG (group IV) and 2.5 g/kg potassium citrate + 1 % EG (group V). Blood and urine were drawn for biochemical analysis, and the liver and kidneys were prepared for total lipid peroxides, calcium content, and histological analysis. The hydromethanol RC extract supplementation helped reduce kidney and liver lipid peroxides to normal levels in rats with EG-induced CaOx lithiasis. In comparison to the control group, the extract decreased renal and urinary calcium contents, decreased the size and number of CaOx calculi in the kidneys, and significantly increased citrate excretion without affecting volume, pH, or urinary oxalate concentrations. The authors concluded that the traditional medicine rosehip extract could prevent and possibly eliminate preexisting kidney stones [63].

Ashtiyani et al. (2013) investigate the beneficial effects of rosehip ethanol extract on histological damages, oxidative stress, and functional disturbances induced by bilateral renal ischemia and reperfusion. The blood and urine samples were drawn for biochemical analysis, and kidney tissues were subjected to microscopic study for histological damages. Compared to the control group, *Rosa canina* group presented low blood creatinine and urea concentrations and showed a ferric reducing/antioxidant power level significantly higher, making rosehip ethanol extract a potential protector of kidneys against function disturbances, oxidative stress, and histological damages [64].

Aqueous extract of rosehip was studied against glomerulonephritis by Martirosyan et al. (2021). Anti-glomerular basement membrane antibodies were used to induce nephritis in mice. From day 5 to day 10, the experimental group was fed with rosehip extract (100 mg/kg body weight/day) by oral gavage, while the control group was fed water. On day 11, mice were sacrificed, and disease phenotypes were determined. Flow cytometry of the kidneys was performed on both groups, and blood urea nitrogen and proteinuria were measured. Treatment with rosehip extraction decreased proteinuria, blood urea nitrogen, compared to control group. The researchers of this study concluded that rosehip may be useful in managing glomerulonephritis [65].

### 2.7 Cardioprotective effects

Cavalera et al. (2017) studied the effects of rosehip extracts on atherosclerotic ApoE-null mice. The purpose of this study was to investigate the molecular mechanisms by which rosehip lowers plasma cholesterol and whether it has any protective effects in the vasculature by reducing atherosclerosis and inflammation. The conclusion of the research presents that they discovered that blood pressure and atherosclerotic plaques, as well as oxidized LDL, total cholesterol, and fibrinogen levels, were significantly lower in the rosehip group. Rosehip feeding significantly increased fecal cholesterol content, Ldlr expression in the liver, and the expression of selected reverse cholesterol transport (RCT) genes such as Abca 1, Abcg1, and Scarb1. The scavenger receptor Cd36 and the proinflammatory Il1 genes were significantly downregulated in the aorta compared to CTR mice. Finally, they discovered that rosehip increased nitric oxide-mediated caudal artery dilation. These findings suggest that rosehip is an effective dietary supplement for preventing the formation of atherosclerotic plaques by modulating systemic blood pressure and the expression of RCT and inflammatory genes [66]. In the study by Nasrolahi et al. (2020), the cardioprotective effects of rosehip methanolic extract were evidenced by its ability to modulate the expression of critical markers related to ER (endoplasmic reticulum) stress, including CHOP. This key transcription factor, which promotes apoptosis under severe cellular stress, showed reduced expression following treatment with rosehip extract. The findings demonstrated that the extract significantly reduced the overproduction of reactive oxygen species - a primary cause of oxidative damage and a precursor to ER stress in cardiomyocytes. This reduction in oxidative stress correlated with decreased CHOP levels, indicating diminished ER stress and a lower initiation of apoptosis pathways. These results highlight rosehip extract's potential in mitigating myocardial damage induced by heat stress, primarily through its antioxidative properties and its capability to modulate ER stress pathways [67].

Moreover, the rosehip extract effects along endurance exercise were studied on the expression of P53 and cytochrome C genes in myocardium of rats. RT-PCR analysis was used to extract RNA, synthesize cDNA, and evaluate the P53 and cytochrome C genes in rat myocardium. The results showed that neither endurance exercise or rosehip, alone or in combination, significantly affected the expression of cytochrome C and P53 genes in male rat heart muscle. In addition, endurance exercise and rosehip supplementation had a significant effect on body weight, myocardium weight, and the ratio of myocardium weight to body weight in male rats, both alone and in combination [68].

### 2.8 Hepatoprotective effects

Banan Khojasteh et al. (2017) focused on studying how rosehip extract affects the quantity of biochemical serum components, the amount of liver enzymes, and the histophysiology of diabetic symptoms in rats. According to the study's findings, treatment with an ethanol extract of rosehip significantly lowered the levels of liver enzymes in the treated groups when compared to the diabetic group. This indicated that the alcoholic extract of rosehip had a protective effect on liver tissue against diabetes-related harm. When rosehip extract was administrated, there were no significant pathological alterations of the liver, being relatively healthier than those of the diabetic group [69]. Rosehip was able to reduce the abnormal increase on various variables caused by d-galactose when used in conjunction with the latter. Using a high dose of d-galactose solution or eating meals with a high galactose content could create a background condition for non-alcoholic fatty liver, which could be reduced by crude rosehip extract. Hence, the rosehip qualifies as a hepatoprotective herb [70]. Another study focused on the impact of a rosehip extract on hypercholesterolemic rats' lipid profiles, liver, and thyroid functions. Wistar rats (60 male) were divided into six groups for this experiment: the control group, the hypercholesterolemic vehicle group, the hypercholesterolemia groups receiving either the extract of *Rosa Canina* at doses of 50, 500, or 1000 mg/kg, either atorvastatin at a dose of 10 mg/kg as gavage for 48 days. It was shown that the levels of many liver enzymes, including alanine transaminase (ALT), aspartate aminotransferase (AST), and alkaline phosphatase (ALP), were significantly reduced in the groups treated with the extract at doses of 500 mg/kg and 1000 mg/kg, rosehip presenting healing effects on dyslipidemia and liver protection [71].

Another research focused on the hepatoprotective properties of the hydro-ethanolic fruit extract of rosehip against rat liver damage produced on by carbon tetrachloride (CCl4) [72]. A significant increase in the serum levels of aminotransferase, alanine amino transaminase, alkaline phosphatase, and lipid peroxidation, as well as a decrease in the levels of albumin and total protein, were indicators of hepatotoxicity. Lymphocyte infiltration and central venous congestion were similarly brought on by the injection of CCL4. Treatment with 500 and 750 mg/kg of the hydro-alcoholic fruit extract of rosehip considerably decreased the levels of aminotransferase, alanine amino transaminase, alkaline phosphatase, lipid peroxidation and malondialdehyde that were raised by CCl4 [72] At the recommended dose, the extract also elevated the serum levels of albumin and total protein in comparison to the CCl4 group. Thus, the hydro-alcoholic fruit extract of *R. canina* may had hepatoprotective effects on CCl4-induced liver damage in rats, by lowering oxidative stress [72].

## 3 Applications of rosehip in food and medical industries

With the growing global population and increasing awareness of the connection between nutrition and overall health, the demand for diverse and health-promoting food products has escalated. As such, the food industry is striving to meet the consumer necessities and seeking sustainable sources of valuable ingredients. In this context, rosehip emerges as a potential candidate due to its wide-ranging functionalities. The increasing focus on sustainability and eco-friendly practices in industries has sparked interest in rosehip's potential in applications beyond traditional medicine. In addition to the fruit itself, rosehip by-products hold significant potential for valorization [73]. Its inclusion in functional foods, nutraceuticals, cosmetics, and pharmaceuticals has been explored, showcasing its versatility and economic potential. As a result, value-added foods, or functional foods with higher levels of dietary fiber and antioxidants have evolved, especially in bakery and cereal products like cookies, gingerbread, or noodles. Substitution of flour in a standard cookie recipe with 15 % rosehip powder and 25 % hibiscus powder showed to increase total phenolic content, antioxidant capacity all along high acceptance when it comes to sensory evaluation [74]. Moreover, by-products of rosehip are valorized in cereal products such as noodles. In Turkish noodles, flours from seed of rosehip, grape and pomegranate were added in concentrations of 10, 20 and 30 %. The addition of 10 % of these seed flours presented significant antioxidant activity [73]. It was shown by Vartolomei & Turtoi, (2021) that the substitution of wheat flour with rosehip powder led to a decrease in moisture, protein, and wet gluten content, while increasing ash, fiber, and carbohydrate content, and introducing vitamin C at levels proportional to the amount of rosehip powder used. Compared to the control bread, the bread with the added rosehip powder showed a significant increase in height, volume, specific volume, moisture, acidity, and porosity, as well as a slight decrease in elasticity. The conclusion indicated that rosehip could be a potential substitute for synthetic ascorbic acid in the bakery industry [75].

Corn extrudates enriched with rosehip puree co-product was another object of study for researchers. The study's aim was to evaluate its impact on extrusion parameters, physicochemical properties, and nutritional characteristics. The addition of 5 % and 10 % rosehip puree enriched extrudates with flavonols, carotenoids, vitamin C, folate. The phenolic acids increased from 165.63 μg/g dry weight in the control sample to 173.3 μg/g dry weight for the 10 % rosehip puree samples, while total flavonols have higher values (694.0 μg/g dry weight). Vitamin C content of 56.19 μg/g dry weight in control samples was increased eightfold by the addition of 10 % rosehip puree, while antioxidant activity increased threefold. In addition, rosehip puree improved the physicochemical properties of extrudates [22].

Rosehip products were shown to have applicability in the dairy industry as well. By using a combination of an *in vitro* gastrointestinal digestion/Caco-2 cell culture model activity increased, researchers looked at the impact of bovine or almond milk fortification on the bioaccessibility and intestinal absorption of rosehip infusion phenolics, primarily catechin. The transfer of phenolics through the epithelial cell layer was positively impacted, according to the findings, by the bovine milk matrix. One could draw the conclusion that it is possible to create functional infusion beverages with increased rosehip phenolic stability, bioaccessibility, and absorption effectiveness in formulations with milk matrix [76]. Furthermore, investigations were also conducted on rosehip's impacts on ice cream's mineral content, antioxidant activity, and physical, chemical, and sensory properties. Rosehips were used in ice cream production in varying percentages (0, 5 %, 10 %, and 15 %), with the 15 % rosehip sample displaying the highest antioxidant activity 1555 mg GAE g^−1^ extract, respectively) and vitamin C (246.50 mg 100 g^−1^) of all the ice cream samples. Also, mineral elements (Ca, Mg, S, K, Mn, Fe, Zn) values increased with the addition of rosehip to ice cream [77].

Moreover, rosehip presents applications in meat industry as well, where was most added to products such as sausages. Pork sausages were produced with rosehip polyphenols, sodium nitrite, butylated hydroxyanisole (BHA), and tetrasodium pyrophosphate, added in various amounts and combinations, smoked, and refrigerated for 20 days. Chemical analyses (peroxide value, thiobarbituric acid reactive chemicals, and protein patterns), microbiological analysis (total plate count), and sensory analyses (color, odor, flavor, texture, and taste) were carried out to assess the quality of the sausages. The findings demonstrated that sodium nitrite (0.01 %), BHA (0.005 %), and tetrasodium pyrophosphate (0.3 %) were not as effective at preventing lipid peroxidation in sausages as the treatment with rosehip polyphenols (0.005 %) and sodium nitrite (0.01 %). Polyphenols extracted from rosehip added to pork sausages at a concentration of 0.005 %, along with 0.01 % sodium nitrite, can delay lipid oxidation, protein degradation, microbial growth, and preserve the bright red color, flavor, and taste of smoked pork sausages for up to 20 days of refrigeration storage. Furthermore, it was shown that rosehip polyphenols alone do not inhibit lipid peroxidation, protein degradation, and microbial growth and are unable to maintain colour, flavour and texture of sausages as effectively as associated with sodium nitrite [78]. Also, Armenteros et al. (2013) showed that natural antioxidants from strawberry tree and rosehip in frankfurter sausages in combination with traditional additives (sodium ascorbate and nitrite) enhanced the oxidative stability without modifying the color and texture properties of sausages [79]. In another study, rosehip extract rich in polyphenols and ascorbic acid in combination with sodium ascorbate and sodium nitrite showed the same results as in the ones previously presented, concluding that rosehip can act as a natural antioxidant in meat sausages, but not as a full replacer for traditional additives [80].

Various technological approaches were adapted for the integrated valorization of rosehip fruits in foods and nutraceuticals with potential health benefits. Several technological versions, including jellified products, juices, and a freeze-dried powder containing *Lactobacillus acidophilus*, were developed, and characterized using the rosehip from Romanian spontaneous flora. The phytochemical profile of the corresponding rosehip pulps, without and with enzymatic pretreatment, revealed that epicatechin was the most abundant compound. The pulp was enriched with pectin and inoculated with *Lactobacillus acidophilus* to create a new perspective for rosehip fruit valorization. Furthermore, significant anti-tyrosinase, anti-diabetic, and anti-obesity potential of the powder was suggested when compared to the effect of current medical practice drugs [81].

Rosehip oil along with cinnamon oil was added to the Aloe Vera gel coating with the purpose to maintain quality and extend the shelf life of pomegranate arils [82]. According to the findings of this study, different treatments have a significant impact on the quality attributes of pomegranate arils. The aloe vera and rosehip oil coating reduced ethylene production more than the other coating combinations, while the aloe vera and cinnamon oil coated arils retained more phenolic compounds, reducing the microbial activity. In this case, aloe vera and cinnamon oil was the most affective coating combination, but it can't be neglected that aloe vera and rosehip oil coatings had potential on reducing activity of microorganisms and reducing the ethylene production [83].

Eggs are a common animal-derived food that is inexpensive and high in bioactive substances with high biological value such as polyunsaturated fatty acid (PUFA). However, eggs undergo physiochemical changes that reduce their value during storage. The effect of dietary rosehip meals and flaxseed meals on hens' egg quality characteristics, amino acids, fatty acids, health-related indices, antioxidant capacity, total polyphenols content, and shelf life was investigated Vlaicu et al. (2022). It was shown that eggs that come from hens fed with rosehip and flaxseed presented high essential amino acids, antioxidant amino acids, and sulfur amino acids contents. Moreover, total antioxidant capacity and polyphenol content increased in the groups fed with flaxseed meal and these eggs maintained significantly higher egg quality parameters after storage for 14 and 28 days in the refrigerator (5 °C) and ambient temperature (21 °C), compared with those from the control group [84].

The combination of kojic dipalmitate (KDP), an esterified form of kojic acid, and rosehip oil, an antioxidant and skin-regenerating oil, into nanocarrier systems appears to be a suitable strategy for developing high-performance formulations. The characteristics of the formulations included droplet size, size distribution, pH, density, shape. *In vitro* evaluations of skin permeability, antioxidant potential, and tyrosinase inhibitory action were conducted. The findings demonstrated that nanoemulsions containing 1 and 2 mg/mL KDP had incorporation efficiencies better than 95 %, droplet sizes less than 130 nm, acceptable size distribution, a zeta potential of roughly 10 mV, and good stability during 30 days of chilled storage. The created nanoemulsions with KDP and rosehip oil were suitable for nanoscale properties and were stable when refrigerated. The nanoemulsion with 1 mg/mL KDP showed potential for use in cosmetic formulations for the treatment of melasma because it demonstrated scale-up feasibility, displayed antioxidant and depigmenting activities, and allowed the active ingredient to reach the epidermis without penetrating to deeper layers of the skin [85]. Moreover, Darie-Nita et al. (2021) research focused on developing an ecological packaging PLA-based containing bio-plasticizers and chitosan modified with rosehip seed oil. The physical-mechanical, thermal, barrier, antibacterial, and antioxidant capabilities of the resulting biocomposites have been examined in order to identify the formulations with the best features for making food trays and films for packaging applications. In accordance with the obtained results, the elaborated formulations had tensile strength and flexibility depending on whether their composition is rigid or flexible, as well as antibacterial and antioxidant activity, which may allow for prolonged use for food packaging. The created biocomposites made of PLA, chitosan modified, and additives (rosehip seed oil) displayed enhanced processability as well as physical-mechanical, thermal, water vapor barrier, antioxidant, and antibacterial characteristics suited for application in food packaging [86]. Plant extracts have been proposed as potential eco-friendly alternatives to traditional techniques, particularly physical and chemical procedures, to produce nanoparticles (NPs). In study of Jafarirad et al. (2016), it has been documented that zinc oxide (ZnO) NPs can be biosynthesized using both conventional heating (CH) and microwave irradiation (MI) techniques. Zinc nitrate and a precursor made from the flesh extract of rosehip fruits were used to create stable and spherical ZnONPs. The cytotoxicity of ZnONPs was examined on the A549 cell line, the nanoparticles exhibiting dose-dependent toxicity to cells. Thus, the ZnONPs have the potential for a variety of medical and industrial applications [87].

## 4 Techniques for the isolation of bioactive compounds from rosehip and its by-products

The most essential stage in isolating antioxidants from vegetable materials is the extraction procedures. The technique and the solvent are to be carefully selected for the bioactive compounds recovery while avoiding the unwanted compounds. Traditional procedures involve using basic equipment, resulting in uncertain quality of the extracts, low yields, and prolonged extraction time [88]. Although they have some drawbacks, liquid-liquid and solid-liquid extraction methods are still the most frequently utilized procedures. Traditional techniques have been widely embraced for their simplicity, effectiveness, and versatile usefulness, over a period of time [89]. Progress has been made to enhance extraction efficiency and decrease time through improving these processes.

New technological methods are being optimized to extract polyphenols while minimizing environmental and health negative effects. These methods employ advanced techniques such as microwaves, ultrasounds, pulsed electric field, and enzyme-assisted extractions, as well as pressurized liquid and supercritical fluid extractions to selectively extract desired polyphenols with high yield and low or no use of organic solvents [88]. The amount of extract obtained is strongly influenced by various factors including the duration of the extraction process, temperature, ratio of solvent to sample, number of times the sample is extracted and the type of solvent used. The solubility of the components in the sample is affected by the temperature and time of extraction. Increasing the temperature increases the solubility and facilitates faster extraction due to reduced viscosity and surface tension of the solvent. To get rid of undesired compounds like terpenes, waxes, chlorophylls, and fats, extra steps can be added [89].

Traditional techniques involve conventional solvents such as methanol, ethanol, acetone, diethyl ether, and ethyl acetate, which are often blended with varying levels of water. Using these solvents has multiple drawbacks. Not only can they pose a potential threat to human health, but a leftover solvent can also remain in the end products. As a result, extra purification measures are necessary, which not only consume time but also affect the overall cost of the process [20]. In addition, when pure organic solvents are employed, it is difficult to extract highly polar phenolic acids (such as benzoic and cinnamic acids), necessitating the usage of mixtures like alcohol–water or acetone–water. Nonpolar compounds such as waxes, oils, sterols, and chlorophyll can be extracted from materials using less polar solvents like dichloromethane, chloroform, hexane, and benzene [90].

Table 1 presents a concise summary of representative extraction methods used by researchers to extract bioactive compounds from rosehips and its by-products. These methods encompass various approaches, including conventional solvent extraction, supercritical fluid extraction (SFE), ultrasound-assisted extraction (UAE), and microwave-assisted extraction (MAE), among others.

**Table 1:** Analytical techniques applied to unlock the health-promoting potential and applicability of rosehip and their by-products.

Extraction method	Source	Parameters	Bioactive compounds	Yields	Applications	References
Supercritical fluids extraction	Rosehip fruits and seeds	Pressure: 35 MPa; Temperature: 45 °C Time: 120 min; Supercritical CO_2_ mass flow rate of 0.42 kg/min	Oleoresins; Polyunsaturated fatty acids; Polyphenols; Phytosterols; Carotenoids	1.85 % oleoresins; 15.6 g oil/100 g dw; 6.9 g GAE/100 g dw polyphenols	Functional powders for food and pharmaceutical applications	[91]
Ultrasonic extraction	Rosehip fruits	Ultrasonic power: 300 W; Temperature: 60 °C Time: 30 min;	Phenols; Flavonoids; Anthocyanins; Vitamin C; Vitamin E; Carotenoids	48.57 to 89.07 TA mg/g dw polyphenols; 13.6 RE mg/g dw flavonoids; 23.75 μg/g dw anthocyanins; 15.09 mg/g dw vitamin C; 28.35 μg/g dw vitamin E; 0.37–0.66 mg/g dw carotenoids	Natural source of antioxidants for the food and pharmaceutical industries	[92]
Maceration and ultrasonication	Wild and cultivated rosehip seed	1 h ultrasonic bath; 68 h at room temperature for maceration	Linoleic acid; α-linolenic acid; Oleic acid	24.53–46.68 % linoleic acid; 4.73–12.39 % α-linolenic acid; 3.89–13.82 % oleic acid	Production of functional foods	[93]
Ultrasonication	Pasteurized rosehip	Treatment times: 5, 10, 15 and 30 min	Ascorbic acid; Carotenoids; Phenolics	108 mg/L nectar ascorbic acid; 2585 μg/mL gallic acid equiv.; 24.1 μg β-carotene equivalent/mL	Production of rosehip nectar	[94])
Chemical extraction	Rosehip fruits	30 min of shaking; Centrifugation at 10.000*g* for 5 min	β-carotene; Lycopene; α-tocopherol; Polyphenolic compounds; Triterpenoic acids	373 mg/kg β-carotene; 176 mg/kg lycopene; 72 mg/kg to 226 mg/kg α-tocopherol; 72 mg/kg to 914 mg/kg phenolics; 36–772 mg/kg betulinic acid; 66–1723 mg/kg oleanolic acid; 37–2531 mg/kg ursolic acid	Food and pharmaceutical industries	[24]
Chemical extraction	Dehydrated rosehip powder	Methanol-water 50:50, v/v; 10.000 rpm; 2 min at ultraturax	Phenolic compounds; β-carotene; Lycopene	51.1–31.5 mg GAE/g dw phenolic compounds; 6.7–4.4 mg CE/g dw; 122–24.1 mg/kg fw lycopene; 13–8.4 mg/kg fw β-carotene	Enhanced the bioactive compounds retention during drying processes	[95]
Ultrasound-assisted extraction combined with deep eutectic solvents	Rosehip fruits	Temperature: 25 °C; Time: 25 min	Phenolic compounds;	8.13 mg GAE/g dw	Food, cosmetic, and pharmaceutical industries	[96]

TA-tannic acid; RE-rutin equivalents; GAE-gallic acid equivalents; CE-catechin equivalents; dw-dry weight; fw-fresh weight.

## 5 Conclusions

Rosehips and their by-products have gained significant attention in recent years due to their abundance of bioactive compounds, such as polyphenols, carotenoids, vitamins, tocopherols, essential lipids, and minerals, which are linked to a wide array of health benefits, including anti-inflammatory, antioxidant, anticancer, and hepatoprotective activities. Despite the promising health benefits of rosehips, there is still a lack of research on their bioactive compounds and potential applications in functional products. The primary challenges include variability in the concentration and composition of bioactive compounds due to varying growing conditions, harvest times, and processing methods, which can impact the consistency and efficacy of final products. Additionally, there is a need for standardized methods to assess the bioactivity of these compounds across different studies and applications. The cost and technical complexity involved in advanced extraction and formulation techniques may also limit the scalability and commercial viability of rosehip-based products. Finally, regulatory hurdles and the need for extensive safety and efficacy testing before market entry add another layer of complexity to the development of rosehip-derived therapeutics and functional foods. Further research is required to address these challenges and fully harness the benefits of rosehips in improving human health and well-being.

## 6 Future recommendations

To fully exploit the functional and nutraceutical potential of rosehips, future research should focus on identifying the specific bioactive compounds responsible for the observed health benefits and optimizing their extraction and purification methods. More clinical trials are essential to establish the efficacy and safety of rosehips in functional foods and medical applications. Additionally, the development of novel formulations and delivery systems to enhance the bioavailability and stability of rosehip bioactive compounds is crucial. The use of advanced technologies, such as nanotechnology and microencapsulation, could facilitate the development of innovative rosehip-based products. This interdisciplinary approach will not only showcase the versatility of rosehips in various industries but also promote sustainable directions for cross-sector collaboration and product development. By addressing these recommendations, research can move towards developing effective and safe rosehip products that improve human health and well-being.

## Data availability statement

Data included in article/supp. material/referenced in article.

## CRediT authorship contribution statement

**Oana-Raluca Negrean:** Writing – original draft, Investigation, Conceptualization. **Anca Corina Farcas:** Writing – review & editing, Supervision, Project administration, Investigation, Funding acquisition, Conceptualization. **Silvia Amalia Nemes:** Writing – original draft, Investigation. **Diana-Elena Cic:** Writing – original draft, Investigation. **Sonia Ancuta Socaci:** Writing – review & editing.

## Declaration of competing interest

The authors declare that they have no known competing financial interests or personal relationships that could have appeared to influence the work reported in this paper.

## Abbreviations

AA: Ascorbic acid
ABTS: 2,2′-azino-bis (3-ethylbenzothiazoline-6-sulfonic acid)
ALT: Alanine transaminase
AST: Aspartate aminotransferase
ALP: Alkaline phosphatase
BHA: Butylated hydroxyanisole
BHT: Butylated hydroxytoluene
cDNA: Complementary DNA
CE: Catechin equivalents
COX-1: Cyclooxygenase-1
COX-2: Cyclooxygenase-2
DPPH: 2,2-diphenyl-1-picrylhydrazyl
DW: Dry weight
ED50: Effective concentration at which the absorbance is 0.5
EG: Ethylene glycol
FRAP: Ferric reducing antioxidant power
FW: Fresh weight
GAE: Gallic acid equivalents
HbA1c: Hemoglobin A1c
HDL: High density lipoprotein
HPLC: High performance liquid chromatography
IC50: Inhibitory concentrations 50 % of cells
KDP: Kojic dipalmitate
LDL: Low density lipoprotein
PLA: Polylactic acid
PUFA: Polyunsaturated fatty acid
RC: Rosa canina
RCT: Reverse cholesterol transport
RE: Rutin equivalent
RNA: Ribonucleic acid
ROS: Reactive oxygen species
RT-PCR: Reverse transcription polymerase chain reaction
SDT: Spontaneously diabetic Torii
TA: Tannic acid
TE: Trolox equivalent
